# Hypoxia induces stress fiber formation in adipocytes in the early stage of obesity

**DOI:** 10.1038/s41598-021-00335-1

**Published:** 2021-11-02

**Authors:** Golnaz Anvari, Evangelia Bellas

**Affiliations:** Department of Bioengineering, Temple University, 1947 N. 12th St, Philadelphia, PA 19122 USA

**Keywords:** Biomedical engineering, Mechanisms of disease

## Abstract

In obese adipose tissue (AT), hypertrophic expansion of adipocytes is not matched by new vessel formation, leading to AT hypoxia. As a result, hypoxia inducible factor-1⍺ (HIF-1⍺) accumulates in adipocytes inducing a transcriptional program that upregulates profibrotic genes and biosynthetic enzymes such as lysyl oxidase (LOX) synthesis. This excess synthesis and crosslinking of extracellular matrix (ECM) components cause AT fibrosis*.* Although fibrosis is a hallmark of obese AT, the role of fibroblasts, cells known to regulate fibrosis in other fibrosis-prone tissues, is not well studied. Here we have developed an in vitro model of AT to study adipocyte-fibroblast crosstalk in a hypoxic environment. Further, this in vitro model was used to investigate the effect of hypoxia on adipocyte mechanical properties via ras homolog gene family member A (RhoA)/Rho-associated coiled-coil kinases (ROCK) signaling pathways. We confirmed that hypoxia creates a diseased phenotype by inhibiting adipocyte maturation and inducing actin stress fiber formation facilitated by myocardin-related transcription factor A (MRTF-A/MKL1) nuclear translocation. This work presents new potential therapeutic targets for obesity by improving adipocyte maturation and limiting mechanical stress in obese AT.

## Introduction

Obesity, a disease characterized by excess adipose tissue, is a worldwide epidemic problem and a risk factor for many other costly comorbidities such as type II diabetes, cardiovascular diseases, stroke, and some cancers. The Centers for Disease Control and Prevention (CDC), in 2017–2018, reported the prevalence of obesity in adults was 42.4% in the United States (https://www.cdc.gov/nchs/products/databriefs/db360.htm). Adipose tissue (AT) is a highly vascularized organ that plays a critical role in metabolic function by storing and releasing fatty acids in response to energy needs. This occurs through adipocyte hyperplasia and hypertrophy, in early, normal tissue expansion^1–3^. However, in obesity, adipocyte hypertrophy and lack of hyperplasia, lead to rapid tissue expansion, which is not matched by new vessel formation, resulting in AT hypoxia^4^. As a result, hypoxia inducible factor-1⍺ (HIF-1⍺) accumulates in adipocytes, inducing a transcriptional program that upregulates profibrotic genes and biosynthetic enzymes such as lysyl oxidase (LOX). These factors lead to the excess synthesis and crosslinking of extracellular matrix (ECM) components causing fibrosis that hinders the dynamic ECM remodeling needed for the healthy expansion of AT^5,6^. Although fibrosis is a hallmark of obese AT, the mechanisms and cell types involved in fibrosis are not well studied^7^.

Fibroblasts are major contributors to ECM synthesis in other fibrosis-prone tissues like skin and liver^8,9^. Fibroblasts become activated (known as myofibroblasts), during fibrosis in fibrotic tissues, leading to increased ECM deposition. These myofibroblasts, exhibit increased alpha-smooth muscle actin (α-SMA) expression, increased actin stress fiber formation, and exert contractile forces to their ECM^9^. The lack of oxygen tension, as in hypoxia, can activate fibroblasts resulting in increased α-SMA and more ECM synthesis, further leading to fibrosis^10,11^. However, little is known about the role of fibroblasts in AT fibrosis and ECM remodeling and how they can be affected by microenvironmental cues like hypoxia.

Hypoxia can affect cellular mechanical properties through the ras homolog gene family member A (RhoA)/Rho-associated coiled-coil kinases (ROCK) pathways, leading to actin cytoskeletal remodeling. For example, RhoA/ROCK signaling activation in cancer cells can increase their motility and invasion, or in stem cells can promote upregulation of chondrogenic markers via actin-myosin tension even on a soft substrate^12–15^. Similar to cancer cells and chondrocytes, adipocytes experience a hypoxic environment as part of an obese phenotype. Yet, the effect of hypoxia on the RhoA/ROCK mechanical signaling pathway is not well understood for adipocytes, although a decrease in RhoA/ROCK signaling is implicated in stem cell fate for adipogenic differentiation^15^. Further, lipid droplet accumulation, a feature of adipogenesis and adipocyte maturation, occurs through decreased cytoskeleton tension (i.e., decreased RhoA/ROCK signaling) and increased cortical actin formation^16^.

To investigate the effect of hypoxia on adipocyte-fibroblast crosstalk and on adipocyte mechanical properties, we have developed an in vitro model of AT by encapsulating adipocytes with or without fibroblasts in collagen I hydrogels, since collagen I is a main structural AT ECM component, and exposing the constructs to hypoxia to mimic the early stage of obesity^17,18^. In vitro three dimensional AT models provide a reductionist model in which we can systematically examine the interactions between adipocytes and their microenvironment^19–27^. Interestingly, co-culture with fibroblasts, did not affect adipocyte gene expression and ECM remodeling. However, hypoxia exposure led to a diseased phenotype by inhibiting adipocyte maturation through downregulation of adipogenic genes and inducing actin stress fiber formation. Adipocyte actin stress fiber formation was due to the nuclear translocation of myocardin-related transcription factor A (MRTF-A/MKL1), a mechanoresponsive transcription factor, and could be reversed by ROCK inhibition. This ROCK inhibition also led to an improvement in key adipocyte genes in hypoxic conditions. Therefore, targeting the downstream effectors of hypoxia via mechanical pathways may be a potential target for obesity treatment.

## Results

### Hypoxia develops a diseased phenotype by inhibiting adipocyte maturation

**Figure 1:**
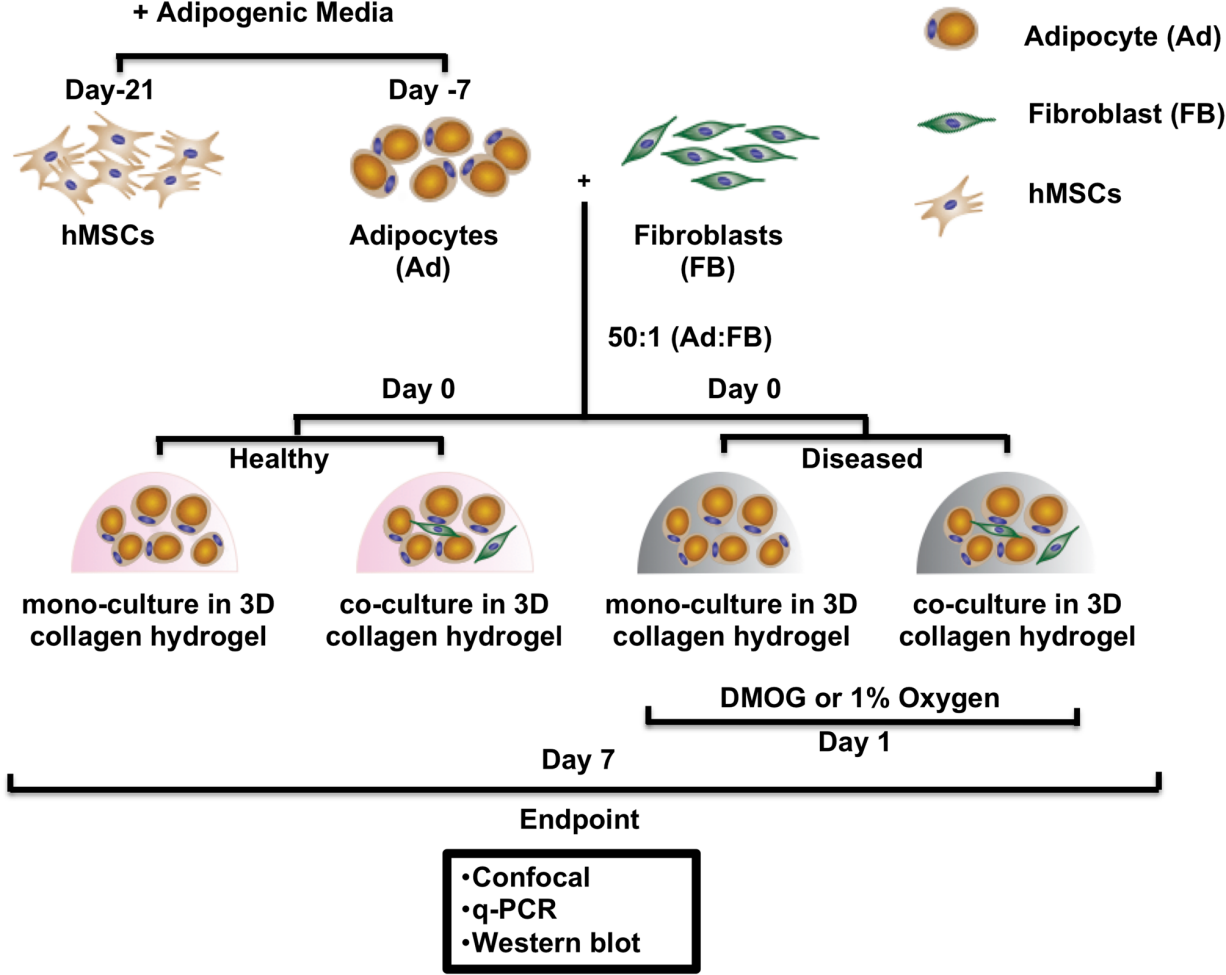
Experimental schematic and workflow. Human mesenchymal stem cells (hMSCs) were expanded in 2D until confluence. To differentiate to adipocytes, adipogenic induction media was added for 7 days. To prepare the 3D constructs, adipocytes were encapsulated in type 1 collagen hydrogels (2 mg/mL) alone or with fibroblasts in a 50:1 ratio with a final seeding density of 8 million/mL. The constructs were maintained in adipogenic maintenance media. Constructs were kept in normoxia to mimic healthy adipose tissue (AT) or in hypoxia to mimic the early stage of obesity. Hypoxia was chemically induced by 1 mM DMOG addition to the media or by culturing in 1% O_2_. After 7 days, AT constructs were sacrificed for endpoint analyses.

**Figure 2:**
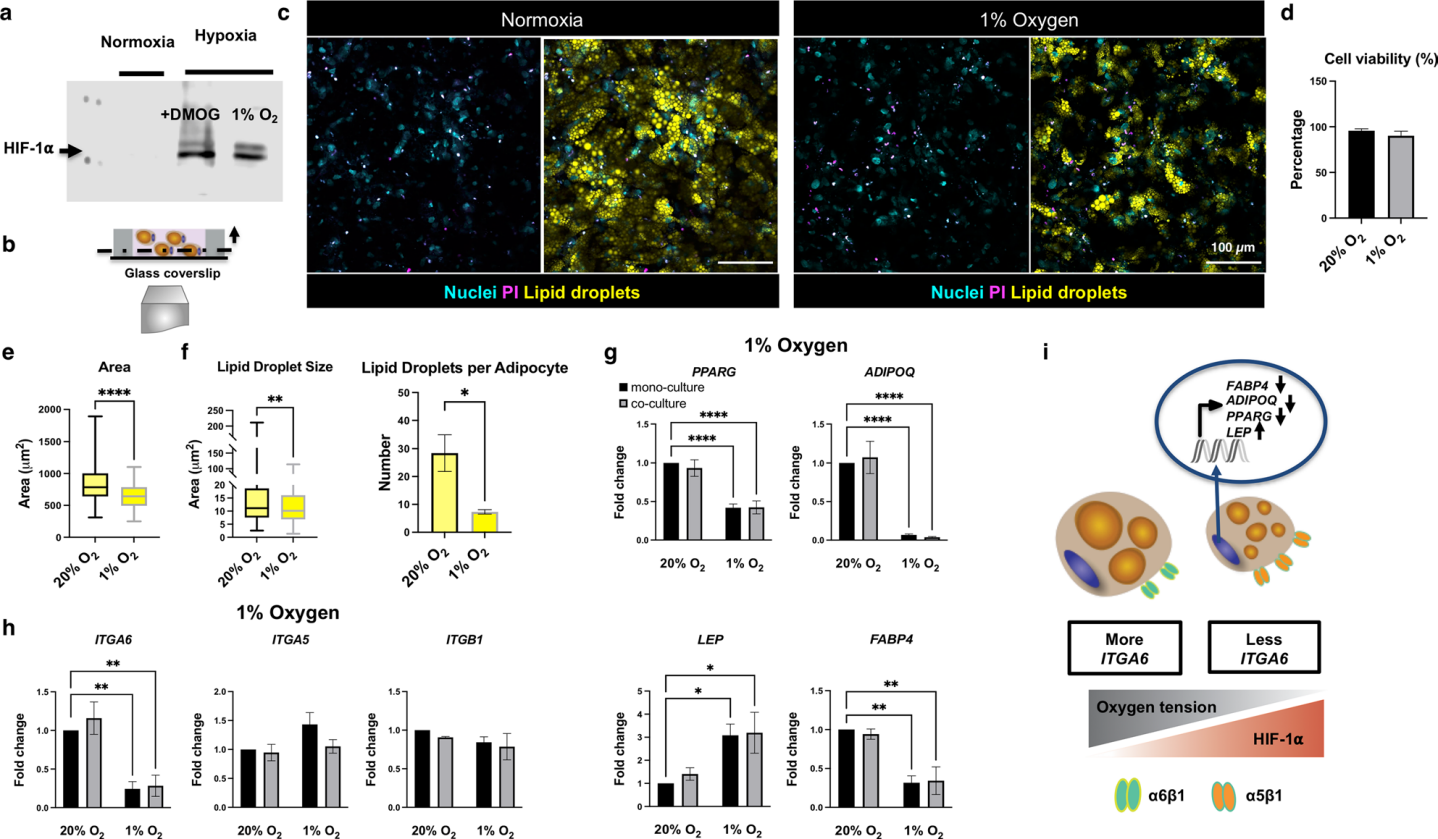
Hypoxia develops a diseased phenotype by inhibiting adipocyte maturation. (**a**) Hypoxia was confirmed by western blot, HIF-1⍺ was present in hypoxic conditions (cropped blot is shown for the mono-culture condition, full length blots are presented in Supplementary Fig. S1). (**b**) Cross-section of AT constructs, fluorescent confocal microscopy images were taken at least 20 µm above the glass coverslip. (**c**) Fluorescent confocal microscopy images of the mono-culture AT constructs in normoxia or 1% oxygen after 7 days stained for Propidium Iodide (PI) to quantify cell viability (scale bar-100 µm). (**d**) Hypoxia did not affect cell viability. (**e**) Adipocyte area was significantly decreased in 1% oxygen, data are presented for the mono-culture condition (n = 4 biological replicates, 14–20 cells per replicate). (**f**) Lipid droplet size and average lipid droplet number per adipocyte were significantly decreased in 1% oxygen, data are presented for the mono-culture condition (n = 3–4 biological replicates). (**g**) Adipocyte gene expression after 7 days, *PPARG*, *ADIPOQ, and FABP4* were significantly downregulated, and *LEP* was significantly upregulated in 1% oxygen (n = 3–8 biological replicates). (**h**) *ITGA6* was significantly downregulated in 1% oxygen (n = 4–8 biological replicates). (**i**) Hypoxia reduces hallmarks of adipocyte maturation through the downregulation of *PPARG, ADIPOQ and FABP4* expression, increasing *LEP* expression and results in smaller and fewer lipid droplets. *ITGA6* gene expression is significantly downregulated in less mature adipocytes. Data are presented as means $\pm$ SEM. Comparisons between groups and statistical analysis were performed using two-way ANOVA with Tukey post hoc test, unpaired t-test or Mann–Whitney test with two-tailed p-values (*p < 0.05, **p < 0.01, ****p < 0.0001).

To create an in vitro AT model, adipocytes, differentiated from hMSCs of healthy donors, were encapsulated with or without fibroblasts within collagen I hydrogels (Fig. 1). Two approaches were used to investigate the effects of hypoxia, dimethylglycine (DMOG) was added to AT constructs (chemically induced hypoxia), or they were exposed to 1% oxygen in a hypoxia chamber. Hypoxia was confirmed by western blot, where HIF-1⍺ was present in both hypoxic conditions. Further, HIF-1⍺ target genes, *LEP* and vascular endothelial growth factor (*VEGF*) were affected in hypoxia (Fig. 2a,g, Supplementary Fig. S1)^28^. There were no differences in adipocyte features between chemically induced hypoxia via DMOG and 1% oxygen groups (Supplementary Fig. S2). Subsequent data sets are shown for constructs in 1% oxygen as a more physiologically relevant model of hypoxia over chemically induced hypoxia.

Adipocyte differentiation and lipid droplet accumulation were confirmed through gene expression and morphological characterization (Fig. 2b,c,e,f, representative confocal images of the mono-culture constructs maintained in 1% oxygen). Hypoxia did not significantly affect cell viability (Fig. 2d). Culturing in 1% oxygen resulted in significantly smaller lipid droplet size and number and cell area (Fig. 2e,f). When exposed to hypoxia, adipocyte specific genes, adiponectin (*ADIPOQ),* peroxisome proliferator-activated receptor gamma (*PPARG)*, and fatty acid binding protein 4 *(FABP4)* were significantly downregulated, whereas leptin (*LEP)* was upregulated (Fig. 2g). The presence of fibroblasts did not affect adipocyte maturation (Fig. 2g,h, Supplementary Fig. S8). It is known that alpha integrin expression shifts from α5 in preadipocytes to α6 in mature adipocytes during adipogenesis . Since decreased adipocyte maturation in hypoxia was observed, integrin α6 (*ITGA6*) and integrin α5 (*ITGA5*) gene expression were quantified. *ITGA6* was significantly downregulated in the hypoxia condition, while no significant changes were seen for *ITGA5* and integrin β1 (*ITGB1*) (Fig. 2h, Supplementary Fig. S2). Altogether, these data demonstrate that, hypoxia reduces hallmarks of adipocyte maturation through downregulating *PPARG, ADIPOQ*, increasing *LEP* expression, and results in smaller and fewer lipid droplets. Furthermore, *ITGA6* gene expression is significantly downregulated in less mature adipocytes (Fig. 2i).

### Hypoxia increases fibronectin, *FN,* and crosslinking enzyme, *LOX*, expression, and results in a distinct fibronectin matrix assembly

**Figure 3:**
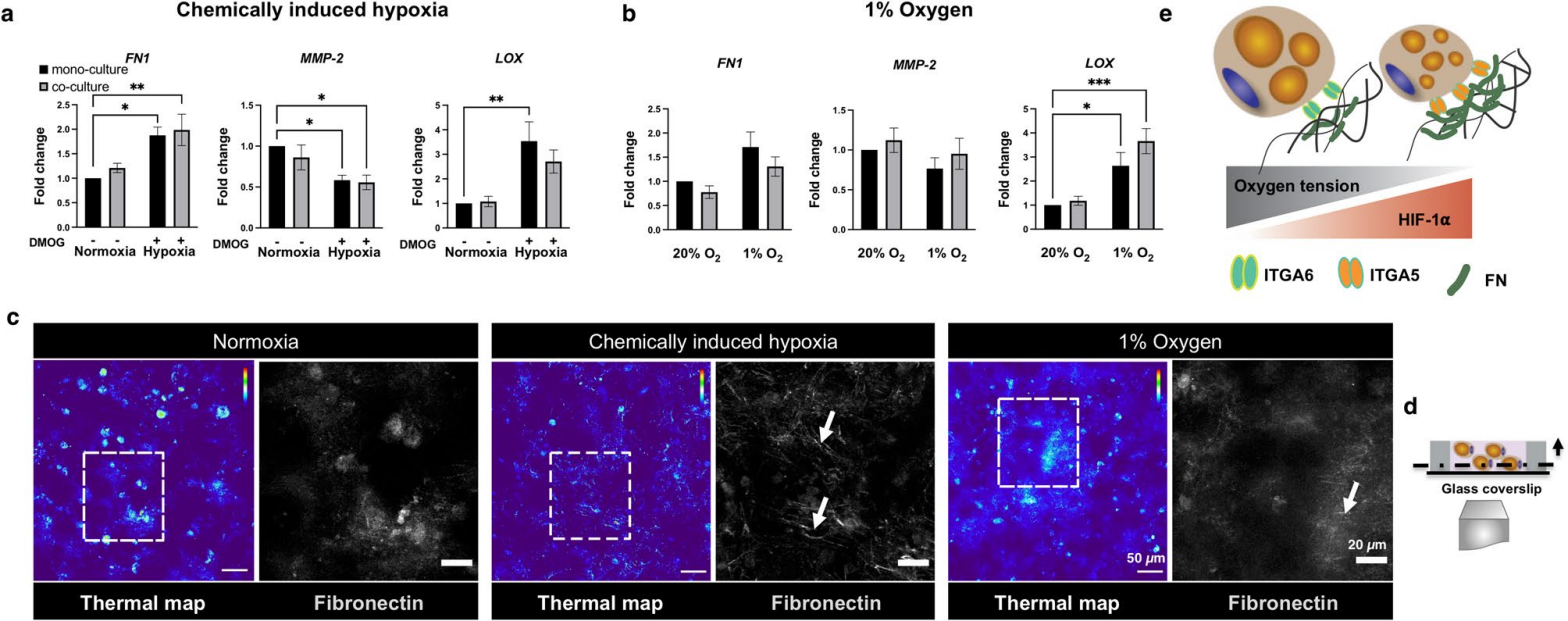
Hypoxia increases fibronectin, *FN,* and crosslinking gene, *LOX*, expression and results in a distinct fibronectin matrix assembly. (**a**) *FN* and *LOX* are significantly upregulated, while *MMP-2* is downregulated in the chemically induced hypoxia (n = 8 biological replicates). (**b**) *LOX* was significantly upregulated in 1% oxygen (n = 8 biological replicates). (**c**) Fluorescent confocal microscopy images of the mono-culture AT constructs in normoxic (thermal map on the left and magnified view of ROI on the right) or hypoxic conditions (thermal map on the left and magnified view of ROI on the right) after 7 days (arrows point to the fibrillar structure of fibronectin, scale bar-50 µm, magnified images-20 µm). (**d**) Cross-section of AT constructs, fluorescent confocal microscopy images were taken at least 20 µm above the glass coverslip. (**e**) Hypoxia increases *FN* and *LOX* expression, and remodels fibronectin matrix into a fibrillar structure. Data are presented as means $\pm$ SEM. Comparisons between groups and statistical analysis were performed using two-way ANOVA with Tukey post hoc test (*p < 0.05, **p < 0.01).

Integrins provide the main molecular links attaching cells to ECM^29^. To further investigate the effect of hypoxia on integrins and their interactions with ECM, we assessed gene expression for the ECM proteins that bind to integrins α6 and α5, *LAMA4, and FN,* respectively*. FN* expression was significantly upregulated, and matrix metalloproteinase-2 (*MMP-2*), known to degrade fibronectin, was significantly downregulated in the chemically induced hypoxia, but not in 1% oxygen. In addition, *LOX* expression, known to increase deposition and assembly of fibronectin fibrils^30,31^, was significantly upregulated in both hypoxic conditions (Fig. 3a,b, Supplementary Fig. S5).

Fluorescent confocal microscopy of the constructs for fibronectin revealed that adipocytes can assemble fibronectin into a fibrillar structure in both hypoxic conditions, while a punctuate fibronectin organization was observed in normoxia. However, the fibrillar structure was more prominent in the chemically induced hypoxia group (Fig. 3c,d, Supplementary Fig. S3, representative confocal images of the mono-culture constructs). These results suggest that hypoxia increases *FN* and *LOX* gene expression and promotes a fibrillar fibronectin structure (Fig. 3e).

### Hypoxia induces stress fiber formation through MKL1 nuclear translocation

**Figure 4:**
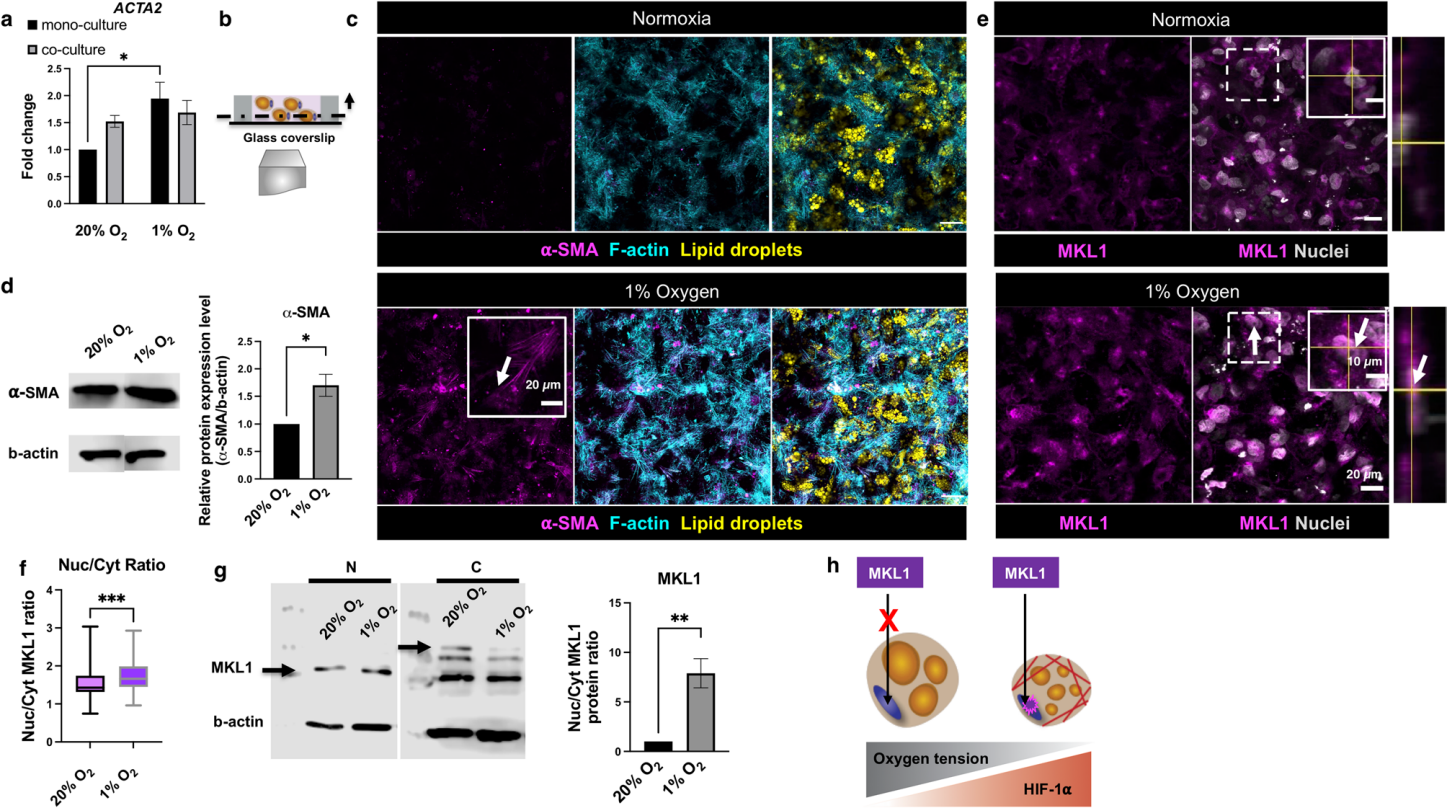
Hypoxia induces stress fiber formation through MKL1 nuclear translocation. (**a**) *ACTA2* was significantly upregulated in hypoxia (n = 8 biological replicates). (**b**) Cross-section of AT constructs, fluorescent confocal microscopy images were taken at least 20 µm above the glass coverslip. (**c**) Fluorescent confocal microscopy images of the mono-culture AT constructs after 7 days in normoxia (top left panel) and 1% oxygen (bottom left panel), arrows point to ⍺-SMA signal and stress fiber morphology (scale bar-50 µm, insets-20 µm). (**d**) ⍺-SMA protein expression was significantly increased in 1% oxygen (data are presented for the mono-culture condition, cropped blot is shown, full length blot is presented in Supplementary Fig. S5). (**e**) Fluorescent confocal microscopy images of the mono-culture AT constructs and the orthogonal view in normoxia (top right panel) and 1% oxygen (bottom right panel), arrows point to MKL1 signal localization (scale bar- 20 µm, insets- 10 µm). (**f**) Nuclear to cytoplasmic ratio for MKL1 was significantly increased in 1% oxygen (n = 5 biological replicates, 20 cells per replicate). (**g**) MKL1 nuclear (N) to cytoplasmic (C) protein ratio was significantly increased in 1% oxygen, arrows point to the quantified MKL1 protein bands (data are presented for the mono-culture condition, n = 3 biological replicates, cropped blot is shown, full length blot is presented in Supplementary Fig. S6). (**h**) Hypoxia induces actin stress fiber formation via MKL1 nuclear translocation. Data are presented as means $\pm$ SEM. Comparisons between groups and statistical analysis were performed using two-way ANOVA with Tukey post hoc test or unpaired Mann–Whitney test with two-tailed p-values (*p < 0.05, **p < 0.01, ***p < 0.001).

Fibronectin fibrillar assembly is known to be mechano-regulated via the cell-mediated contractile forces^32^. These contractile forces are partly related to increased actin stress fiber formation within the cells. Dysfunctional adipocytes have more filamentous actin as they interact with their fibrotic environment. Therefore, we hypothesized that distinct fibronectin matrix assembly and lack of maturation observed in hypoxia (Figs. 2, 3) are related to actin cytoskeletal remodeling. To investigate the effect of hypoxia on actin cytoskeletal remodeling, we examined stress fiber formation in adipocytes. In this model, upon exposure to hypoxia, actin alpha 2 (*ACTA2*), a gene that encodes actin stress fibers (α-SMA), was significantly upregulated (Fig. 4a,b, Supplementary Fig. S4). The presence of actin stress fibers was further confirmed through confocal images and protein expression (Fig. 4c,d, representative confocal images of the mono-culture constructs maintained in 1% oxygen, Supplementary Fig. S5). To further determine the mechanical pathway that results in enhanced α-SMA expression in hypoxia, MKL1, a transcriptional factor translocated to the nucleus during activation of the RhoA/ROCK pathway, and regulates α-SMA expression was explored^33,34^. In normoxia, MKL1 expression was greater in the cytoplasm, in contrast to 1% oxygen, where MKL1 expression was greater in the nucleus. (Fig. 4e, representative confocal images of the mono-culture constructs maintained in 1% oxygen, Supplementary Fig. S6). The nuclear to cytoplasmic distribution ratio for MKL1 was significantly greater in 1% oxygen (Fig. 4f). The greater nuclear to cytoplasmic ratio in 1% oxygen was further confirmed through protein expression (Fig. 4g). Together, these data support that hypoxia induces actin stress fiber formation in adipocytes with MKL1 nuclear translocation (Fig. 4h).

### Y27 treatment partly attenuates hypoxia effects by inhibiting MKL1 nuclear translocation

**Figure 5:**
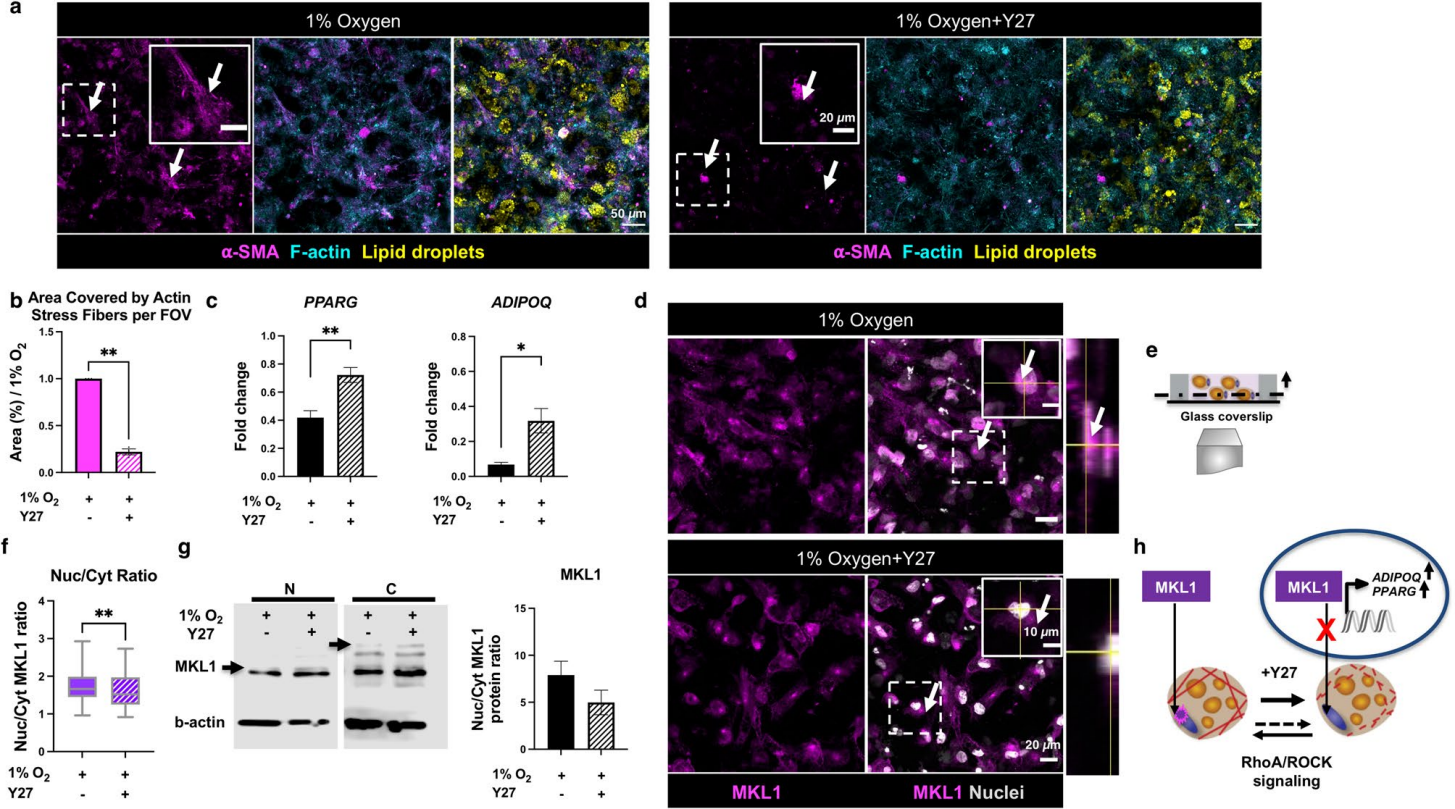
Y27 treatment partly attenuates hypoxia effects by inhibiting MKL1 nuclear translocation. (**a**) Fluorescent confocal microscopy images of the mono-culture AT constructs after 7 days in 1% oxygen without (top left panel) and with (top right panel) Y27 treatment, arrows point to ⍺-SMA signal (scale bar-50 µm, insets-20 µm). (**b**) Actin stress fiber morphology was changed after Y27 treatment (n = 3 biological replicates). (**c**) Adipogenic (*ADIPOQ, PPARG*) genes were partly rescued towards a normoxic phenotype after Y27 treatment (n = 4–8 biological replicates, data are normalized to mono-culture samples maintained in 20% oxygen). (**d**) Fluorescent confocal microscopy images of the mono-culture AT constructs and the orthogonal view in 1% oxygen before and after Y27 treatment, arrows point to MKL1 signal localization (scale bar-20 µm, insets-10 µm). (**e**) Cross-section of AT constructs, fluorescent confocal microscopy images were taken at least 20 µm above the glass coverslip. (**f**) The nucleus to cytoplasm ratio for MKL1 was significantly decreased after Y27 treatment (n = 5 biological replicates, 20 cells per replicate). (**g**) MKL1 nuclear to cytoplasmic protein ratio in 1% oxygen and after Y27 treatment, arrows point to the quantified MKL1 protein bands (n = 3 biological replicates, cropped blot is shown, full length blot is presented in Supplementary Fig. S6). (**h**) Y27 treatment disrupts actin stress fibers due to MKL1 nuclear translocation inhibition, and this process is regulated via RhoA/ROCK signaling pathway. Data are presented as means $\pm$ SEM. Comparisons between groups and statistical analysis were performed using unpaired Mann–Whitney test with two-tailed p-values (*p < 0.05, **p < 0.01).

To determine the molecular pathway that supports stress fiber formation and MKL1 translocation in hypoxia, the RhoA/ROCK mechanical pathway was explored by exposure to Y-27632 (Y27), a biochemical inhibitor of ROCK. After Y27 treatment, the α-SMA signal was present, but the defined stress fiber morphology was not visible, confirming that inhibiting ROCK disrupts stress fiber formation (Fig. 5a,b,e, representative confocal images of the mono-culture constructs maintained in 1% oxygen). Furthermore, the inhibition of ROCK by Y27 led to an upregulation in adipogenic related genes, *PPARG* and *ADIPOQ* (Fig. 5c). Y27 treatment results in decreased MKL1 expression in the nucleus as well as nuclear to cytoplasm distribution ratio, confirming the possible role of ROCK in MKL1 translocation to the nucleus. MKL1 protein expression further confirmed decreased nucleus to cytoplasmic ratio after Y27 treatment (Fig. 5d,f,g, Supplementary Fig. S6). Overall, we demonstrated that RhoA/ROCK signaling pathway regulates actin stress fiber formation and MKL1 nuclear translocation within adipocytes in hypoxia (Fig. 5h).

## Discussion

This study sought to develop an in vitro AT model to study adipocyte-fibroblast crosstalk in healthy (normoxia) and diseased (hypoxia) states. Further, we used this model to investigate the effect of hypoxia as the early stage of obesity on RhoA/ROCK signaling pathway in adipocytes. Multiple cell types in AT contribute to ECM synthesis during fibrosis, such as adipocyte progenitors as well as adipocyte crosstalk with other cells, including macrophages^35–38^. Fibroblasts and activated fibroblasts (myofibroblasts) are sources of ECM production in other fibrosis-prone tissues; therefore, we co-cultured adipocytes with fibroblasts to determine if they played a role in the observed AT diseased phenotype. The ratio of adipocytes to fibroblasts (50:1) was selected to match the ratio found in human AT^39^. We did not observe any significant differences between mono- and co-culture conditions in the assays performed. Further, our results indicated that fibroblasts did not go under myofibroblastic activation under hypoxic conditions. This confirms previous studies demonstrating that hypoxia can inhibit the activation of fibroblasts through inhibition of pathways that lead to α-SMA activation, like SMAD independent pathways and RhoA GTPase dependent actin polymerization (Supplementary Fig. S7)^40–42^. In this crosstalk model, fibroblasts were not activated directly through hypoxia or via adipocyte crosstalk. Therefore, we concluded that hypoxia mainly affects adipocytes through a decrease in their adipogenic regulation (i.e., PPARG) and downstream changes to their mechano-sensitive pathways while other fibrotic stimuli such as transforming growth factor-β (TGF-β) and platelet-derived growth factor (PDGF) may be needed to trigger fibroblast activation^43^.

In the current study, we focused on hypoxia as an early feature of an obese AT. Our results demonstrated that hypoxia creates a diseased model by affecting both adipocytes and their ECM. Hypoxia inhibited maturation was confirmed through smaller and fewer lipid droplets, smaller adipocyte size, and downregulation of *PPARG* and *ADIPOQ,* and upregulation of *LEP*. *PPARG* is the master regulator of adipogenesis, and it is known to upregulate other adipocyte related genes, such as adiponectin (*ADIPOQ*). Similarly, leptin is also an adipokine and a hallmark of adipogenesis. However, leptin is abnormally increased in obesity. Therefore, taken together, downregulation of *PPARG* and *ADIPOQ* and upregulation of *LEP* expression in the constructs exposed to hypoxia indicates a diseased adipocyte phenotype.

Adipocyte differentiation and maturation are, in part, regulated through integrin expression, where integrin α5β1 attachment to fibronectin is replaced by α6β1 attachment to laminin during adipogenesis^44–46^. We found *ITGA6* was significantly downregulated in hypoxia, which supports the earlier results that adipocyte maturation is reduced in hypoxia. While we did not observe transcriptional level changes for *ITGA5*, there are several routes to post-transcriptional integrin α5 regulation, including changes to integrin activation state or shuttling to the cell surface. Interestingly, here we noted differences in the hypoxia groups, where in chemically induced hypoxia, fibronectin (*FN*)*,* the α5β1 integrin binding partner, was upregulated, and at the protein level was organized in a more fibrillar manner. Further, the enzyme which degrades fibronectin, *MMP-2*, was downregulated in chemically induced hypoxia. Taken together, this shows that adipocytes in hypoxia are further inhibited in maturation by increased FN expression and organization with limited matrix remodeling by MMP-2, while also limiting the a6 attachments to their ECM and potentially favoring existing α5 integrins. Determining the α5 integrin activity of adipocytes in hypoxia may increase our understanding of the temporal and functional effects regulating FN organization and adipocyte mechanical pathways.

This study has shown that the methods used to generate hypoxia can lead to differences in outcomes. Given that hypoxia is often confirmed by HIF-1⍺ expression by immunoblot, it was important to note that while both chemically induced hypoxia and 1% oxygen led to similar accumulation of HIF-1⍺ (Fig. 2a). However, the use of a hypoxia chamber to induce hypoxia may be a less stable hypoxic environment, as the constructs are intermittently briefly exposed to normoxia during media exchanges, while chemically induced hypoxia groups are not.

Adipogenesis and adipocyte maturation are dependent on actin cytoskeletal remodeling. This remodeling process involves stress fiber disruption and cortical actin formation to facilitate lipid accumulation^16^. The inhibited maturation we observed in hypoxia is partly related to increased stress fiber formation in adipocytes exposed to hypoxic conditions. In obesity, adipocytes become metabolically dysfunctional due to a fibrotic phenotype, including enhanced expression of cytoskeletal, ECM, and focal adhesion genes^47,48^. MKL1, a mechanoresponsive transcription factor, is a known regulator of myofibroblast differentiation that controls the expression of cytoskeletal and ECM genes such as α-SMA (*ACTA2*); some collagens, as well as *PPARG*^40,41^. MKL1 is prohibited from translocating into the nucleus within the healthy adipocytes due to active actin remodeling (polymerization) and interaction with globular (G)-actin^49–52^. However, when MKL1 is translocated to the nucleus due to RhoA/ROCK activity, it can decrease *PPARG* and increase α-SMA (*ACTA2*) expression, similar to the results observed in hypoxia (Figs. 2g, 4a).

Therefore, disrupting actin stress fibers (depolymerization) by adding Y27 to the constructs enhanced adipocyte related (*ADIPOQ, PPARG*) gene expression, reversing in part, the observed diseased phenotype (Fig. 5c)^52^.

A contractile cell phenotype is known to contribute to fibronectin organization. Previous studies have demonstrated that increased α-SMA and MKL1 expression in myofibroblast enabled those cells to assemble the extrinsic fibronectin to a fibrillar matrix^32,53–55^. This agrees with our findings, where in constructs exposed to hypoxia, we observed increased contractile gene expression, *ACTA2,* which enables these cells to assemble fibronectin molecules into a fibrillar structure due to these applied contractile forces (Figs. 3c, 4a).

**Figure 6:**
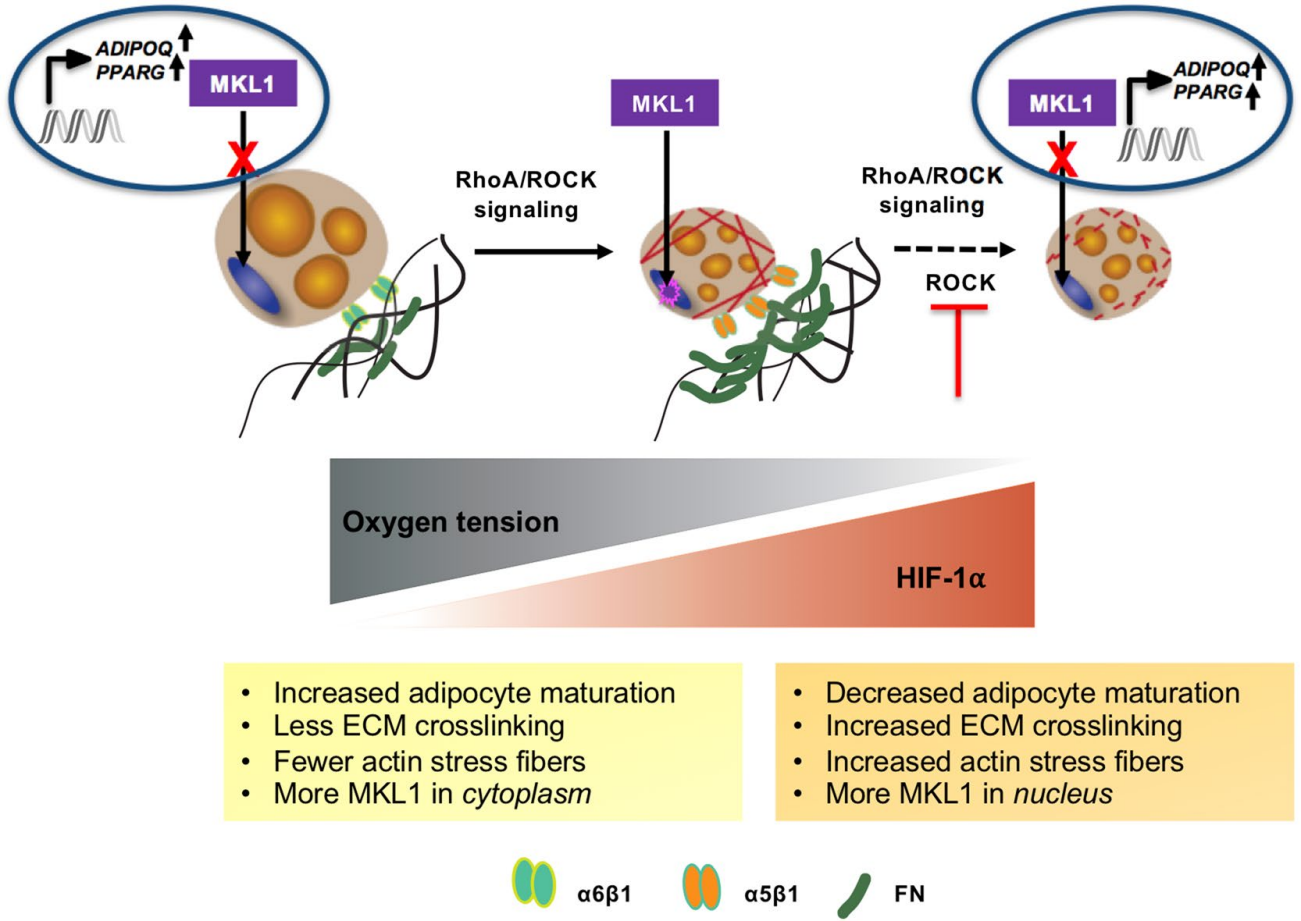
Summary overview. Hypoxia creates a diseased phenotype by (1) inhibiting adipocyte maturation via downregulating adipogenic (*ADIPOQ, PPARG*), and *ITGA6* gene expression, and (2) inducing actin stress fiber formation through MKL1 translocation to the nucleus. MKL1 nuclear translocation is regulated via RhoA/ROCK signaling pathway and inhibiting ROCK attenuates hypoxia effects.

Overall, this study illustrates the role of hypoxia as an early hallmark of obesity in creating a diseased environment not only by affecting adipocyte maturation but also their mechanical properties. Hypoxia affects mechanical properties by inducing stress fiber formation resulting from MKL1 translocation to the nucleus. Therefore, hypoxia and its downstream mechanical pathways, including MKL1 that is regulated via RhoA/ROCK signaling pathway, can be used as a potential target for treating the early stage of obesity (Fig. 6).

## Materials and methods

### Cell culture

Human bone marrow-derived mesenchymal stem cells (hMSCs) were isolated from bone marrow purchased from Lonza Walkersville Inc. (Walkersville, MD, Cat# 1 M-105) and expanded in low glucose Dulbecco’s Modified Eagle’s medium (DMEM, Sigma-Aldrich) supplemented with 10% fetal bovine serum (FBS, Sigma-Aldrich) and 1% penicillin/streptomycin (Sigma-Aldrich) in 2D until confluency. Primary human dermal fibroblasts (HDFB) from neonatal foreskin (Clonetics) were cultured in DMEM with 4.5 g/L glucose supplemented with 10% FBS, 1% Penicillin–Streptomycin, 1% L-glutamine (Cytiva) and used at passages 16–18. Cells were maintained at 37 °C and 5% CO_2_.

### Adipogenic induction of hMSCs

Adipogenic induction media was added when hMSCs reached confluency at passage 4. The basal media used for differentiation composed of DMEM/F12 (Corning) supplemented with 3% FBS and 1% penicillin/streptomycin. The induction media supplements consist of 2 µM rosiglitazone (Cayman Chemical), 500 µM 3-isobutyl-1-methylxanthine (Sigma-Aldrich), 1 µM dexamethasone (Acros Organics), 33 µM biotin (Alfa Aesar), 17 µM D-calcium pantothenate (TCI America), and 20 nM of insulin (Sigma-Aldrich). The hMSCs underwent differentiation for seven days before encapsulation. After encapsulation, the induction media was replaced with media without rosiglitazone and 3-isobutyl-1-methylxanthine, referred to as maintenance media.

### Polydimethylsiloxane cast preparation

Polydimethylsiloxane (PDMS; Sylgard 184, Dow Corning) mixture, the weight ratio of base to curing agent at 10:1, was cast at 100 °C oven for 45 min to fabricate 1 mm thick sheets. The Cured PDMS was cut into squares, and 5 mm holes were made using a 5 mm biopsy punch (AcuPunch). To attach PDMS to the glass coverslip, the surface of both was activated by plasma ashing (PE25W, PlasmaEtc) for 1 min. The assembled devices were then treated with 0.01% (v/v) poly-L-lysine (PLL, Sigma-Aldrich) for 1 h, rinsed thoroughly in deionized (DI) water, and then treated with 1% (v/v) glutaraldehyde (Electron Microscopy Sciences) for 30 min to enhance collagen adhesion. Devices were then rinsed thoroughly in DI water again and ultraviolet (UV) sterilized for 15 min.

### 3D collagen hydrogel preparation and cell encapsulation

Collagen gels were prepared according to an established protocol^56^. In brief, to prepare the collagen hydrogel, type I rat tail collagen stored in acetic acid (Corning) was neutralized with the addition of 1 N sodium hydroxide (NaOH, Fisher Scientific), 250 mM 2-[4-(2-hydroxyethyl) piperazin-1-yl] ethanesulfonic acid (HEPES, Fisher Scientific), 5% (w/v) sodium bicarbonate (NaHCO_3_, Fisher Scientific), 10X Medium199 (Sigma-Aldrich) and water to the final concentration of 2 mg/mL, while maintained on ice. The neutralized collagen solution was mixed with either adipocytes or fibroblasts alone (mono-culture) or the mixture of two cell types (co-culture) in a 50:1 ratio with a final seeding density of 8 million/mL. Collagen droplets with a volume of 30 μL were dispensed into the wells created on the PDMS/glass coverslip device, followed by 30 min of incubation at 37ºC to allow for collagen crosslinking. Fresh maintenance media was added to the wells post-crosslinking and was replenished every 2–3 days.

Constructs were kept either in normoxic conditions or, after 24 h, switched to hypoxic conditions. Hypoxia was induced chemically with the addition of 1 mM HIF-Hydroxylase inhibitor (dimethylglycine, DMOG) (EMD Millipore) dissolved in dimethyl sulfoxide (DMSO, Millipore Sigma) or by placing constructs in 1% oxygen within a hypoxia chamber (Billups-Rothenberg, Inc.) (Fig. 1).

To reduce cytoskeletal tension, Y-27632 (Y27), a pharmacologic ROCK inhibitor (Tocris Biosciences) dissolved in DMSO, was added to the maintenance media every 48 h, at a final concentration of 10 µM.

### Gene expression

RNA was collected from 2–3 pooled constructs per condition using Trizol (Ambion) and stored at -80ºC until needed. Chloroform (Acros Organics) phase separation technique was used to isolate RNA, and the RNA was precipitated in 50% isopropanol (Fisher Chemical). After that, it was washed twice with 75% ethanol and resuspended in diethylpyrocarbonate (DEPC)-treated water (Invitrogen). Total RNA reverse transcription and Real-time PCR (RT-PCR) was performed with High-Capacity cDNA Reverse Transcription Kit (Applied Biosystems) and Power Up SYBR Green Master Mix (Applied Biosystems), respectively. Primers were custom designed (Integrated DNA Technologies). Samples were run in triplicate using an Eppendorf Mastercycler and StepOnePlus PCR system (Applied Biosystems). Fold change in relative gene expression levels was determined using the 2^-ΔΔCt^ method.

### Protein expression

Protein extracts from constructs were obtained by either lysing the cells in 2X laemmeli sample protein buffer (Bio-Rad) supplemented with 1 mM Dithiothreitol (DTT, Sigma-Aldrich) or by radioimmunoprecipitation assay (RIPA) buffer supplemented with phosphatase inhibitor cocktail that contains 250 mM sodium fluoride, 50 mM sodium orthovanadate, 50 mM Sodium pyrophosphate decahydrate, and 50 mM β-glycerophosphate (Sigma-Aldrich). To separate nuclear and cytoplasmic fractions, AT constructs were incubated in hypotonic buffer (20 mM Tris–HCl, 10 mM NaCl, 3 mM MgCl_2_), vortexed and subjected to centrifugation (1699 rcf) and the supernatant was collected as a cytoplasmic fraction. The remaining pellet was resuspended in a cell extraction buffer, vortexed in 10 min intervals for 30 min and subjected to centrifugation (20,817 rcf) and the supernatant was collected as the nuclear fraction.

Proteins were separated by gel electrophoresis using 8% or 12% SDS-PAGE gel and then transferred onto a nitrocellulose membrane (Immobilon-P). After blocking and probing with antibodies, the detection was performed using horseradish peroxidase (HRP, Bio-Rad) conjugated anti-rabbit secondary antibody (1:3000, Cat# 170–6515, RRID:AB_11125142) and enhanced chemiluminescence reagent (Bio-Rad), and the signal was visualized using C-DiGit Blot Scanner.

For protein detection, the following primary antibodies were used: rabbit anti-HIF-1⍺ (1:100, Cell Signaling Technology Cat# 3716, RRID:AB_2116962), anti-MKL1 (1:1000, Proteintech Cat# 21,166–1-AP, RRID:AB_2878822), and rabbit anti-beta actin (1:1000, Cell Signaling Technology Cat# 4970, RRID:AB_2223172), as a housekeeping protein.

### Visualization by immunofluorescence and cell staining

For immunofluorescence analysis, constructs were fixed with 4% (v/v) paraformaldehyde (Thermo Scientific) for 20 min at room temperature (RT) and then permeabilized with 0.1% (v/v) Triton X-100 (Sigma-Aldrich) for 20 min before blocking with 3% (w/v) bovine serum albumin (BSA, Fisher Scientific). To detect collagen I, α-SMA, and MKL1, the following primary antibodies were used at 1:200 dilution: mouse anti-collagen I antibody (Thermo Fisher Scientific Cat# MA1-26771, RRID:AB_2081889), mouse anti-alpha smooth muscle actin (Thermo Fisher Scientific Cat# MA5-11547, RRID:AB_10979529), and rabbit anti-MKL1 (Proteintech Cat# 21166–1-AP, RRID:AB_2878822). To pre-label rat tail collagen, 5-carboxy x Rhodamine succinimidyl ester was used at the final concentration of 1 µg/mL. To assess cell viability, AT constructs were incubated with Propidium iodide (PI, 1:100, Alfa Aesar) followed by fixation with 2% formaldehyde.

Constructs were co-stained for Hoechst 33342 (Thermo Scientific, 1:1000), BODIPY 493/503 (Invitrogen, 1:100), and Alexa Fluor 647 phalloidin (Invitrogen, 1:200) for 45 min at RT, to visualize nuclei, lipid droplets, and actin, respectively, and stored in PBS in 4 °C until imaged.

### Fluorescent confocal microscopy and image analysis

Images were acquired with a laser scanning confocal mode of the hybrid confocal-multiphoton microscope (Olympus FluoView FV1200) using 10X (UPLXAPO10X, 0.4 NA, Olympus) or 30X (UPLSAPO30XSIR, 1.05 NA, Olympus) oil immersion inverted objectives with four main laser units (405, 488, 543, and 635 nm.), and four photomultiplier (PMT) detectors. Z-stacks (thickness ~ 50 µm, step-size: 5 µm, 20 µs/pixel, 1024 × 1024) were captured at least 20 µm above the glass coverslip to ensure visualized cells were in a 3D environment and not in contact with the glass coverslip surface (Figs. 2b, 3d, 4b, and 5e). Brightness and contrast were applied equally to the experimental conditions when appropriate to improve signal and to reduce background.

Acquired images were imported to ImageJ for further analysis. Lipid droplet diameter and number were quantified by an ImageJ Plugin, “MRI_Lipid Droplets Tool” (http://dev.mri.cnrs.fr/projects/imagej-macros/wiki/Lipid_Droplets_Tool). Adipocyte area was calculated by specifying adipocyte area using the area selection tool in ImageJ, and quantifying the area using the measurement tool. Since the adipocyte seeding ratio is high (8 million/mL), to ensure that one cell at the time was measured, image analysis was performed slice by slice such that selected cells are not overlapping. To clarify cell boundaries, we have counterstained the samples with phalloidin to visualize actin. To quantify MKL1, slices from the stacks were z-projected using the Max Intensity method. The nucleus to cytoplasm ratio for MKL1 was calculated using the “RawIntDen” measurement tool, and the intensity value was normalized to the nucleus and cytoplasm area. To quantify the α-SMA area, the α-SMA channel was converted to a binary display, and the percentage area of the signal in the field of view (FOV) was calculated with the measurement tool. Collagen cluster thickness was quantified by using “BoneJ” macro in ImageJ. In brief, BoneJ macro fits spheres along the collagen fibers after a threshold is applied to the image and calculates the thickness as the average largest circle that can fit within the fibers^57,58^. A thermal map for fibronectin was created by pseudo coloring the fluorescent confocal images with thermal lookup table (LUT) in ImageJ (Supplementary Fig. S3).

### Statistics

GraphPad Prism 8 (GraphPad Software Inc.) software was used to perform all statistical analyses. All experiments were conducted with at least three biological replicates and 15–20 cells per biological replicates for image quantification. The distribution of each data set was analyzed, and the D'Agostino-Pearson test (α =  0.05) was performed to test for normality. Statistical comparisons between two experimental groups were performed using two-tailed Student’s t-test or Mann–Whitney test for non-normally distributed data when appropriate. Comparisons among more groups were performed using two-way analysis of variance (ANOVA) with Tukey post hoc testing. All graphs are presented as mean ± standard error of the mean (SEM) unless otherwise stated. Significance was determined according to *p < 0.05, **p ≤ 0.01, ***p ≤ 0.001, and ****p ≤ 0.0001.

## Supplementary Information

Supplementary Information.

## Data Availability

The datasets generated and analyzed during the current study are available from the corresponding author upon reasonable request.
