# Supplementary material for: Hypoxia induces stress fiber formation in adipocytes in the early stage of obesity
Source: Sci Rep. 2021 Nov 2;11:21473. doi: 10.1038/s41598-021-00335-1 (PMC8563745; doi:10.1038/s41598-021-00335-1)
Supplement: Supplementary file 1 — Supplementary Information. [file 41598_2021_335_MOESM1_ESM.docx]

**Supplementary Information**

**Hypoxia Induces Stress Fiber Formation in Adipocytes in the Early Stage of Obesity**

Golnaz Anvari^1^, Evangelia Bellas^1*^

^1^Department of Bioengineering, Temple University, 1947 N. 12th St, Philadelphia, PA 19122, USA

**Corresponding Author**

E-mail: [Evangelia.bellas@temple.edu](mailto:Evangelia.bellas@temple.edu)

**This PDF file includes:**

Supplementary Figs. S1 to S7


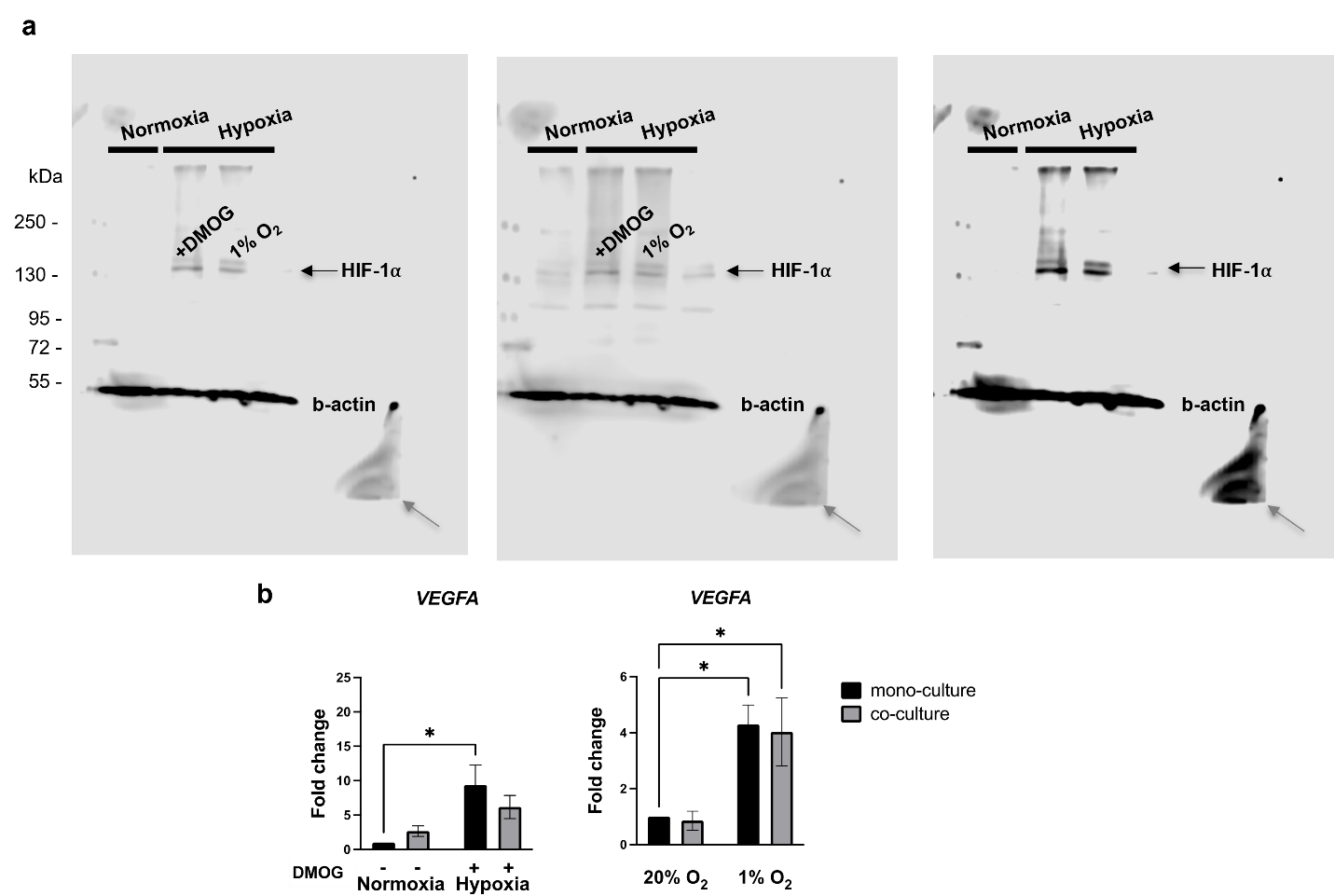


**Supplementary Figure S1. Full length blots for HIF-1⍺ and its target gene, *VEGFA*.** Full length blots for HIF-1⍺ in the mono-culture AT constructs presented with different intensities, black arrows point to HIF-1⍺ bands and grey arrows point the edge of the blot. (b) *VEGF,* a direct target of HIF-1⍺, was significantly upregulated in hypoxic conditions (n=4-7 biological replicates)*.* Data are presented as means $\pm$ SEM. Comparisons between groups and statistical analysis were performed using two-way ANOVA with Tukey post hoc test (*p<0.05). Note: Images are selected from different intensity settings in C-DiGit Blot scanner software which does not alter raw data. Images are not further manipulated to adjust brightness and contrast.

**Supplementary Figure S2. Adipogenic related and integrin gene expression for constructs exposed to chemically induced hypoxia.** (a) Adipocyte gene expression after 7 days, *PPARG* and *ADIPOQ* were significantly downregulated, and *LEP* was significantly upregulated in constructs exposed to chemically induced hypoxia (n=6-8 biological replicates). (b) *ITGA6* was significantly downregulated in constructs exposed to chemically induced hypoxia (n=4-8 biological replicates). Data are presented as means $\pm$ SEM. Comparisons between groups and statistical analysis were performed using two-way ANOVA with Tukey post hoc test or unpaired t-test with two-tailed p-values (*p<0.05, ****p<0.0001).

**
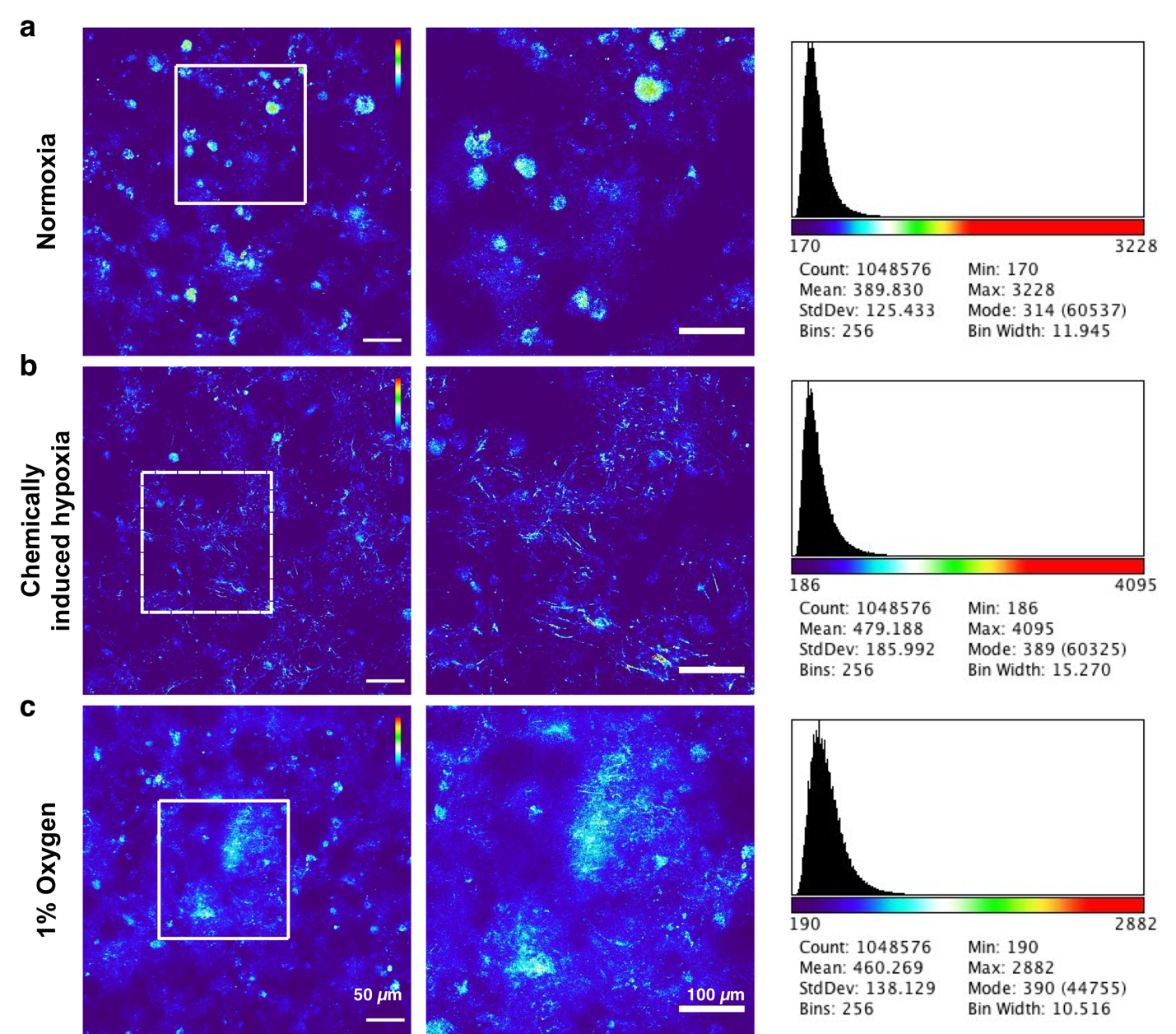
**

**Supplementary Figure S3**. **Thermal maps and LUT histograms for fibronectin confocal microscopy images.** (a) Normoxia, (b) Chemically induced hypoxia, and (c) 1% oxygen, scale bar-50 µm, and magnified images-100 µm.


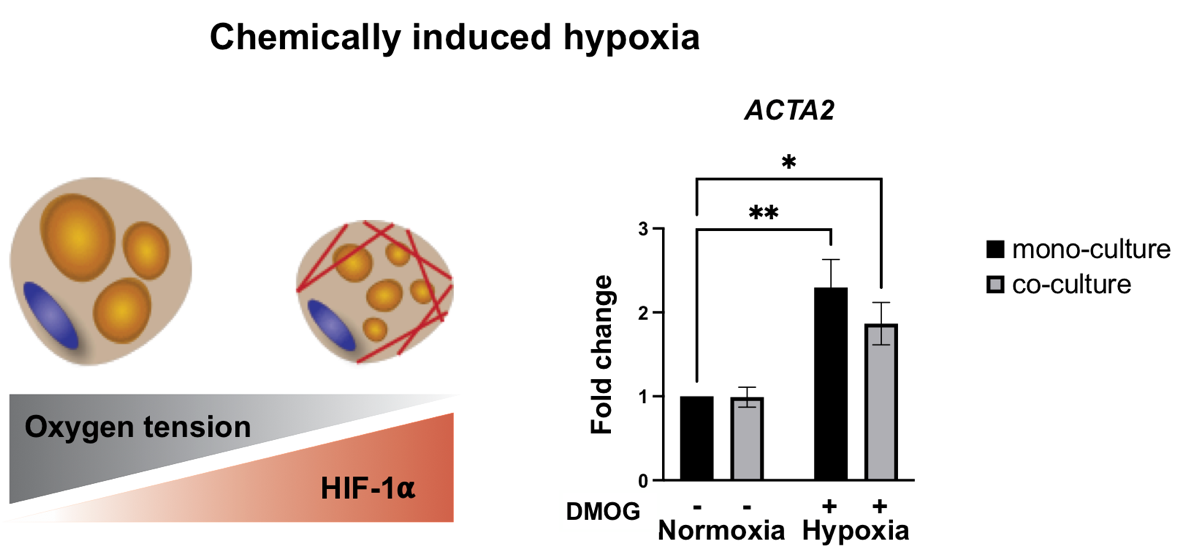


**Supplementary Figure S4. *ACTA2* gene expression for constructs exposed to chemically induced hypoxia.**  *ACTA2* gene expression was significantly upregulated in constructs exposed to chemically induced hypoxia (n=8 biological replicates). Data are presented as means $\pm$ SEM. Comparisons between groups and statistical analysis were performed using two-way ANOVA with Tukey post hoc test (*p<0.05, **p<0.01).


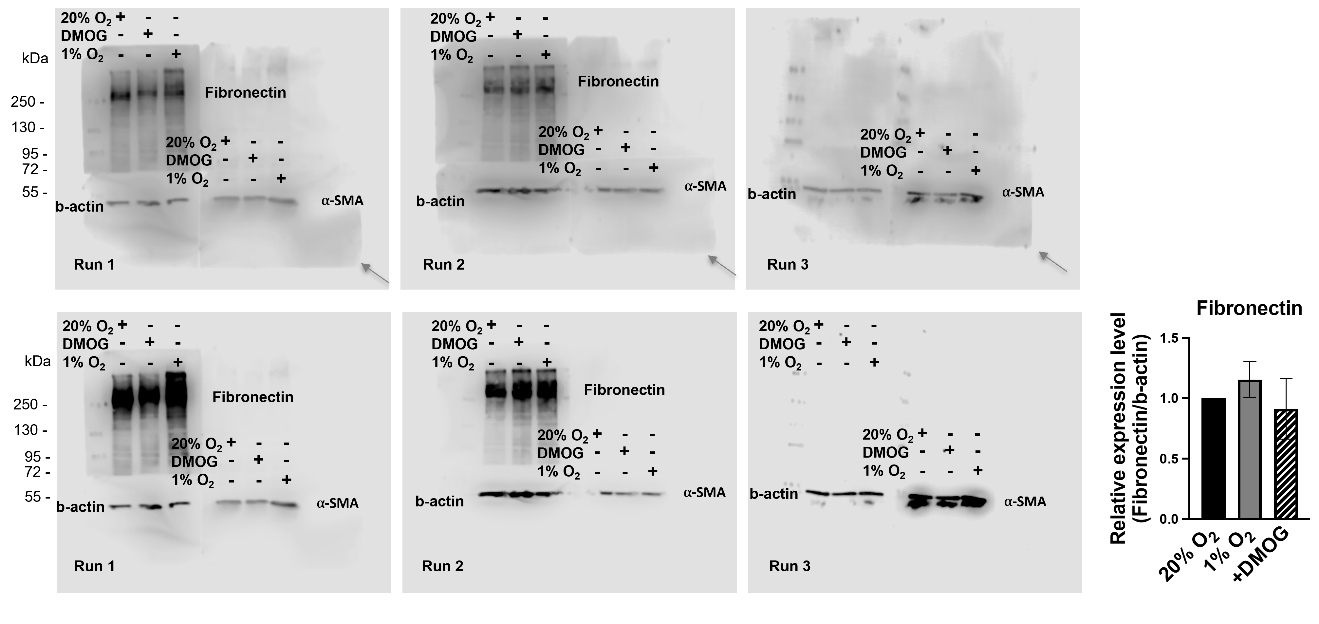


**Supplementary Figure S5. Full length blots for fibronectin and ⍺-SMA protein expression.** Fibronectin and ⍺-SMA protein expression in normoxia, chemically induced hypoxia (+DMOG), and 1% oxygen for the mono-culture condition, with two different intensities (low intensity- top row, high intensity- bottom row) for each biological replicate (n=3), grey arrows point to blot edge. Data are presented as means $\pm$ SEM. Comparisons between groups and statistical analysis were performed using one-way ANOVA or unpaired t-test with two-tailed p-values. Note: Images are selected from different intensity settings in C-DiGit Blot scanner software which does not alter raw data. Images are not further manipulated to adjust brightness and contrast.


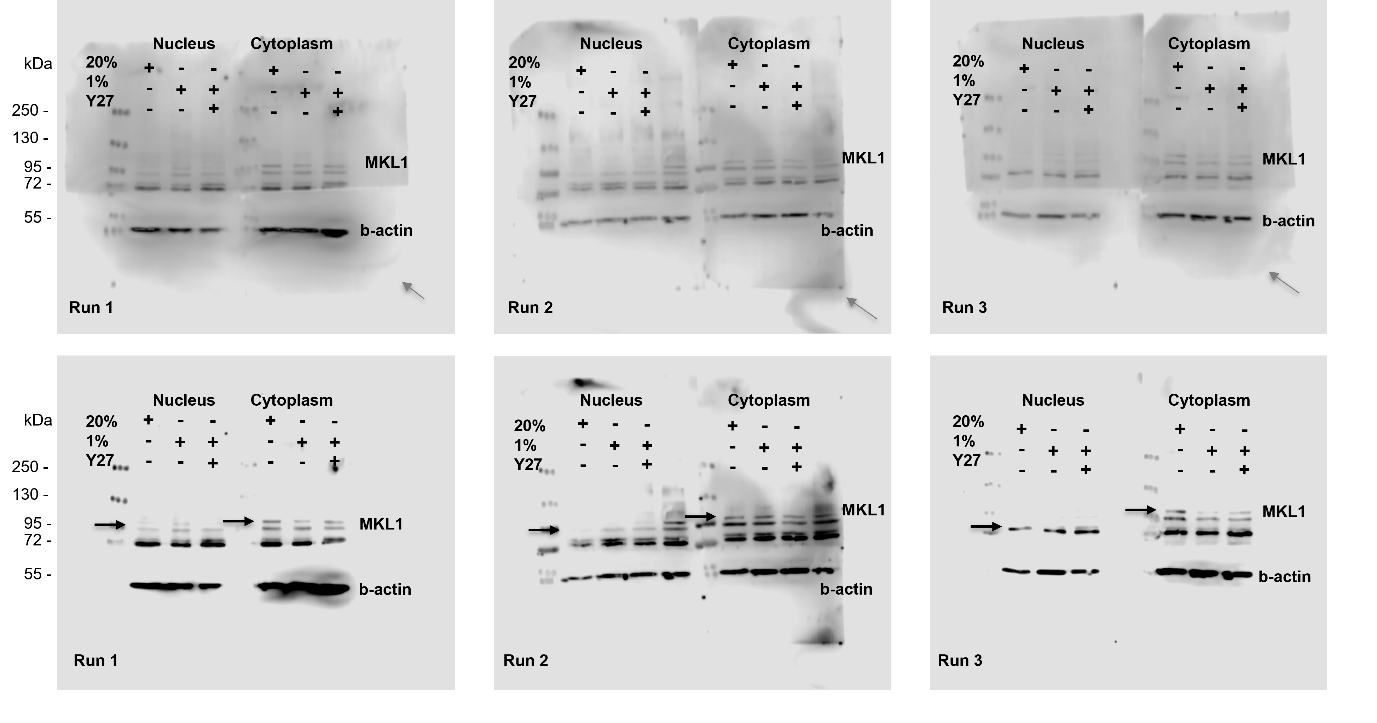


**Supplementary Figure S6. Full length blots for MKL1 nuclear and cytoplasmic protein expression.** MKL1 nuclear and cytoplasmic protein expression in normoxia, 1% oxygen and 1% oxygen+Y27 for the mono-culture condition presented two different intensities (low intensity- top row, high intensity- bottom row) for each biological replicate (n=3), black arrows point to selected MKL1 bands for quantification, grey arrows point to the blot edge. Note: Images are selected from different intensity settings in C-DiGit Blot scanner software which does not alter raw data. Images are not further manipulated to adjust brightness and contrast.


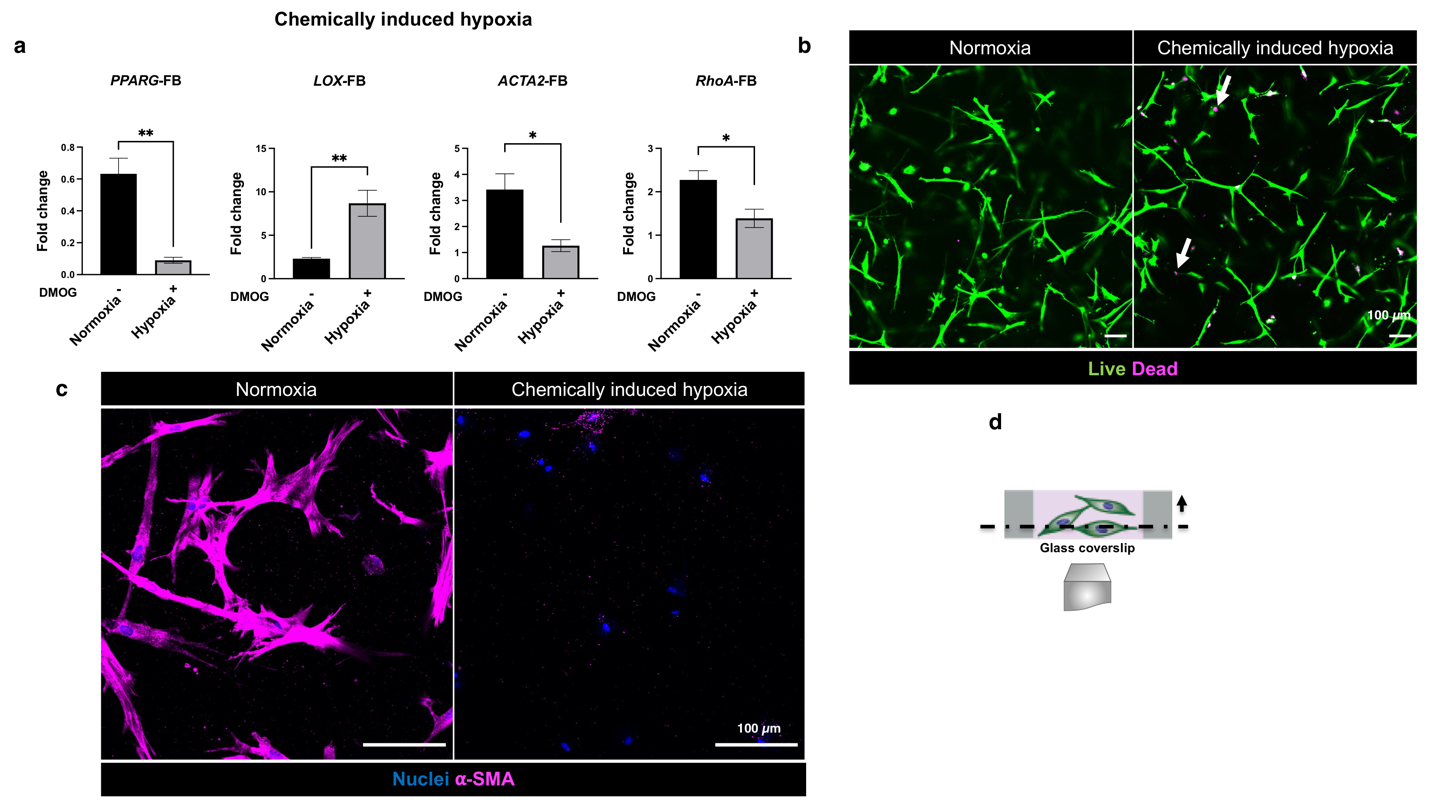


**Supplementary Figure S7. Fibroblasts (FBs) do not undergo myofibroblastic activation in chemically induced hypoxia.** (a) *PPARG, ACTA2, RhoA* were significantly downregulated in chemically induced hypoxia, however *LOX* (a direct HIF-1⍺ target) was significantly upregulated (n=4 biological replicates, data are normalized to adipocyte mono-culture maintained in normoxia). (b) Live/dead staining for FBs in normoxia and chemically induced hypoxia, arrows point to dead cells (scale bar-100 µm). (c) ⍺-SMA signal was not present in chemically induced hypoxia (scale bar-100 µm). (d) Cross-section of FB constructs, fluorescent confocal microscopy images were taken at least 20 µm above the glass coverslip. Data are presented as means $\boldsymbol{\pm}$ SEM. Comparisons between groups and statistical analysis were performed using unpaired t-test with two-tailed p-values (*p<0.05, **p<0.01).
